# The effect of non-steroidal anti-inflammatory drugs on matrix metalloproteinases levels in patients with osteoarthritis

**DOI:** 10.31138/mjr.28.3.133

**Published:** 2017-09-29

**Authors:** Maria Efstathiou, Loukas Settas

**Affiliations:** A.H.E.P.A. Hospital/First Internal Medicine Clinic, Thessaloniki, Greece

**Keywords:** metalloproteinases, NSAIDs, osteoarthritis

## Abstract

**OBJECTIVE:**

The objective of this study is to determine and comparatively evaluate the effects of three different non-steroidal anti-inflammatory drugs on the levels of metalloproteinases MMP-1, MMP-3 and MMP-8, as well as on their tissue inhibitor TIMP-1, in patients suffering from idiopathic osteoarthritis. The effect of these drugs on the articular cartilage and the probable use of MMPs and TIMP-1 as markers of disease and treatment was also investigated.

**METHODS:**

Thirty-six patients with OA were selected and allocated to three groups on the basis of their disease location. All patients received anti-inflammatory treatment with special selective COX-2 inhibitors, i.e. celecoxib, meloxicam, aceclofenac. Each drug was given to every patient for three months following a randomized order of administration. Serum levels of MMP-1, MMP-3, MMP-8 and TIMP-1, and ratios MMP-1/TIMP-1, MMP-3/TIMP-1, MMP-8/TIMP-1 were measured before and after treatment.

**RESULTS:**

The use of aceclofenac resulted in no significant variation in either MMPs concentration and MMPs/TIMP-1 ratio. This outcome concerns the three groups and the 36 patients that form them. After all patients had received all three NSAIDs, MMPs and TIMP-1, these parameters were compared to their initial and final median values. A significant reduction in MMP-3 was found so in all OA patients as in the group of knee OA patients.

**CONCLUSIONS:**

1. Of the MMPs studied, MMP-3 levels were found to be significantly reduced after NSAIDs treatment. Therefore, serum MMP-3 levels in OA patients could be proven to be a useful evaluating marker of treatment on the cartilage level. 2. No significant differences were observed among NSAIDs administered with regards to their effect on MMPs and TIMP-1 concentration.

Osteoarthritis (OA), the most prevalent disease of articular joints,^1^ is a group of overlapping distinct diseases, which may have different etiologies. The disease processes affect not only the articular cartilage, but involve the entire joint leading to the articular cartilage degeneration with fibrillation, fissures, ulceration and full thickness loss of the joint surface.^2^ The aetiology of OA is not yet completely understood, but could be considered to be the result of a complex system of interacting mechanical, biological, biochemical and enzymatic mechanisms. The final result is the failure of chondrocyte to maintain a homeostatic balance between matrix synthesis and degradation.^3^

A great deal of attention is focused on identifying the proteases responsible for the initial occurrence of matrix digestion. Current knowledge indicates that matrix metalloproteinases (MMPs) are very important for the chondrolytic processes which contribute to the degenerative changes in OA cartilage.^4–7^

Matrix metalloproteinases are a group of Zn2+ dependent extracellular enzymes that are active at neutral PH and play a key role in a normal and pathological tissue remodelling, such as development, wound healing, atherosclerosis, tumor invasion and arthritic diseases.^8–10^ The whole group of MMPs can be divided into subclasses such as collagenase, gelatinases, stromelysins and membrane type MMPs according to their specificity to degrade the extracellular matrix protein components, including the collagens, proteoglycans, fibronectin and laminin.^3^ In our trial, members of the first three groups of MMPs have been found to be elevated. Moreover, in OA, MMP-1 and MMP-8 are located predominantly in the superficial and upper intermediate layer, whereas MMP-13 is found mostly in the lower intermediate and deep layers of cartilage.^11^ From the group of stromelysins, only stomelysin-1 (MMP-3) appears to be involved in OA.

MMP biologic activity is controlled physiologically by specific tissue inhibitors of metalloproteinases (TIMPs) or by their proteolytic activation. In OA tissue, there is an imbalance between the amounts of TIMPs and MMPs resulting in an increased level of active MMPs.^12^

Many factors are implicated in the complex regulation of MMP production including proinflammatory cytokines, such as interleukin-1 (IL-1) and tumor necrosis factor (TNF-a) and evidence strongly supports the concept that IL-1b and TNF-a are the major catabolic systems involved in the destruction of joint tissues in OA.^13–15^

The ideal drug therapies for OA could have four aims, namely to relieve symptoms, to improve function, to offer protection from any further cartilage destruction and/or to reverse the morphological changes.

So far, drug therapies in OA can be classified as either symptom modifying OA drugs (SMOADS) or disease modifying OA drugs (DMOADs).^16^,^17^ Although several drugs have been used in OA therapy and have demonstrated properties of “chondroprotection”, until now no drug could be classified as DMOAD, that is to demonstrate the ability to prevent, delay progression of, or reverse morphological changes.^18^ Hence, it is very important to assess treatments used until now for OA management from the point of their action in articular cartilage.

NSAIDs are commonly used for the treatment of OA and are recognised to effectively relieve symptoms and reduce inflammation. Their mechanisms of action are not yet fully understood and in particularly their action in cartilage remain unclear and under investigation. The effect of NSAIDs on animal cartilage matrix metabolism has been studied over a number of years, and Dingle and collaborators have divided NSAIDs in three categories with respect to their in vitro action upon anabolic functions of OA cartilage.^19^ So far very few in vivo studies and a very small number of clinical studies have been carried out to evaluate the role of NSAIDs in cartilage metabolism.^20,21^ This study was designed to measure the levels of three MMPs and specifically of MMP-1, MMP-3 and MMP-8 and their tissue inhibitor, TIMP-1 in patients with OA and to comparatively evaluate the effects of treatment with three NSAIDs, i.e. celecoxib, meloxicam and aceclofenac, on the articular cartilage.

## PATIENTS AND METHODS

### Study Population

During the period January 2001 – February 2002, 82 patients with OA according to the American College Rheumatology (ACR) criteria^22–25^, were examined in the Department of Rheumatology of 1st Internal Medicine Clinic Medical School of Aristotle University of Thessaloniki, Greece. In all patients, demographic and clinical data were described and laboratory examinations, X-Rays and MRI of the joints affected were carried out.

Subjects had to meet the following eligibility criteria: male or female aged 5075 years; a diagnosis of primary osteoarthritis of at least 3 months in symptom duration prior to screen; fulfilling ACR criteria for symptomatic OA of the knee, hands and hip; documented anteroposterior radiographic evidence of tibio-femoral OA within past 12 months (grade II or III according to the Kellgren and Lawrence scale).^26^ American Rheumatism Association (ARA) functional class I, II or III^27^; evaluation of pain between 50 mm and 80 mm on the 100 mm visual analogue scale (VAS).

Exclusion criteria were: known history of hypersensitivity to NSAIDs, aspirin, COX-2 inhibitors or paracetamol; active seizure disorder; secondary causes of arthritis including septic arthritis, inflammatory joint disease, articular fracture, major dysplasias or congenital abnormality, ochronosis, acromegaly etc.; disease of the spine or other lower extremity joints of sufficient degree to affect the index knee; significant prior injury to the index joint within the last 12 months; lower extremity surgery within the last 6 months; history of active upper and lower gastrointestinal (GI) disease; history of congestive heart failure, coronary artery disease, uncontrolled hypertension, renal artery stenosis and malignancy; use of anticoagulants or antiplatelet aggregation agents (except low aspirin dose, i.e. 100 mg per day); any other clinical or laboratory found at screen which was valued as clinically significant (e.g. levels of transaminases 1,5x upper limit of normal, high levels of creatinine, major depression etc.).

From the 82 patients, who were screened, 58 patients with OA were enrolled. During the study, of the eligible 58 patients, five chose not to participate in the study, three withdrew due to adverse events and four were lost to follow up. Therefore 36 patients completed the study and were divided into three groups according to their disease location taking into account the fact that OA comprises a heterogenous group of diseases, each of which is characterized by specific clinical and pathological findings:^28^

Group A: Patients with knee OA
Group B: Patients with hand OA (with Heberden’s and Bouchard’s nodes)
Group C: Patients with hip OA

Twenty healthy volunteers were used as control.

**Table 1** shows the baseline characteristics of study group according to gender, age and OA location.

**Table 1.** Effect of celecoxib on MMPs and TIMP-1 levels in OA

	**Patients with OA**			
	**knee OA (n=16)**	**hip OA (n=10)**	**hand OA (n=10)**	**total (n=36)**

MMP-1^a^ BT	6,53	4,02	4,92	5,39
MMP-1 AT	6,4	4,27	5,72	5,62
MMP-1/TIMP-1^b^ BT	0,003	0,001	0,002	0,002

MMP-1/TIMP-1 AT	0,003	0,001	0,002	0,002
MMP-3^a^ BT	25,59	24,10^*^	13,46	21,8^*^
MMP-3 AT	15,21	10,91	10,92	12,51
MMP-3/TIMP-1^b^ BT	0,13^*^	0,002	0,006	0,10^*^
MMP-3/TIMP-1 AT	0,008	0,002	0,003	0,005

MMP-8^a^ BT	19,93	23,94	12,68	19,03
MMP-8 AT	11,63	19,36	13,46	14,28
MMP-8/TIMP-1^b^ BT	0,009^*^	0,008	0,006	0,008
MMP-8/TIMP-1 AT	0,005	0,007	0,003	0,006

TIMP-1^a^ BT	225,62	264,00	225,00	236,11
TIMP-1 AT	320,62	275,00	237,00	284,36

* : p<0,01

a : Serum concentrations in ng/ml

b : Ratio

BT: Before treatment

AT: After treatment

### Study design

The study had a randomized, cross-over design. The 36 patients received non-steroidal antiinflammatory drugs (NSAIDs), i.e. celecoxib (a special COX-2 inhibitor), meloxicam and aceclofenac (selective COX-2 inhibitor) according to a randomized order of administration.

Participants were treated with 100 mg celecoxib twice a day, meloxicam 15 mg once a day and aceclofenac 100 mg twice a day and the washout period between each NSAID administration was 15 days.

From the total group of 36 patients, serum samples of MMP-1, MMP-3, MMP-8 and TIMP-1 were obtained and estimated before and after treatment with each NSAID. After patients received treatment with all the three NSAIDs, the same parameters were evaluated according to their mean value before and after treatment independently of the order of administration. The ratios MMP-1/TIMP-1, MMP3/TIMP-1 and MMP-8/TIMP-1 were also evaluated and were compared before and after treatment with each one. Furthermore, it was investigated whether the different order of drug administration could influence the mean value of MMPs and TIMP-1. All the aforementioned comparisons were carried out in all patients even in the subgroups according to OA location so that to investigate different expressions of MMPs could be investigated.

### Measurement of MMPs and TIMP-1

The serum levels of MMP-3, MMP-1, MMP-8 and TIMP-1 were measured using the sandwich enzyme linked immunosorbent assay (ELISA, R & D Systems, Minneapolis, USA), following the manufacturer’s instructions.

Sensitivity of assays were 0,021 ng/ml, 0,009 ng/ml, 0,02 ng/ml and 0,08 ng/ml, respectively. In preliminary experiments, the suitables dilutions of samples were determined (1:1, 1:10, 1:20, 1:100, respectively).

### Statistical analysis

Statistical analysis was carried out using the SPSS ver. 10 Statistic Package (SPSS Inc., Chicago, IL, USA). For the data with normal distribution, paired t-test was used (values at the beginning and end of treatment) while Wilkoxon signed rank sum test was used to analyze matched pairs with abnormal distribution.

In order to compare the changes of values in the same population, 2-way ANOVA with factors for the location of OA and for each NSAID was applied. Data were checked with Mauckly’s test of sphericity. For the case of statistical significance, the Greenhouse-Geisser test was performed.

In order to estimate statistical significance between different drugs or location of OA, one-way ANOVA was used. In the case of abnormal distribution of data, the Kruskal Wallis test was carried out.

The probable correlation between MMPs, TIMP-1 and VAS analogue scale was examined by determining the Spearman rank order correlation coefficient. P values less than 0.05 were considered to be statistically significant.

## RESULTS

### Effect of celecoxib on serum levels of MMPs and TIMP-1

**Figure 1.**
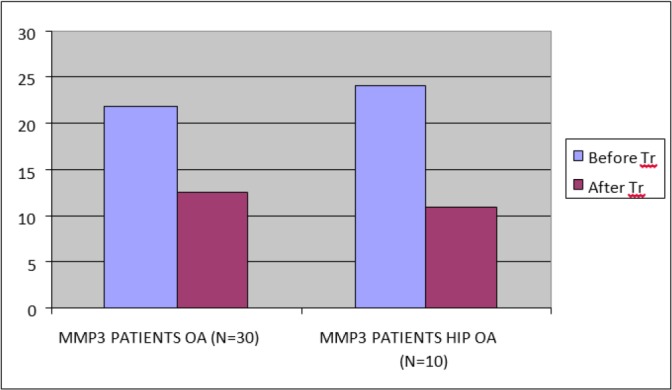
Effect of celecoxib on MMP-3 concentrations in all patients and in hip OA patients

During celecoxib administration, there were no significant differences in mean serum values of MMP-1 and MMP-8 when these were compared before and after treatment for all 36 patients of study (**Table 1**). In contrast to this, the serum levels of MMP-3 were significantly reduced after treatment with celecoxib (p=0,001, **Figure 1**) while the mean value of TIMP-1 was not significantly increased (p>0,05, **Table 1**).

**Figure 2.**
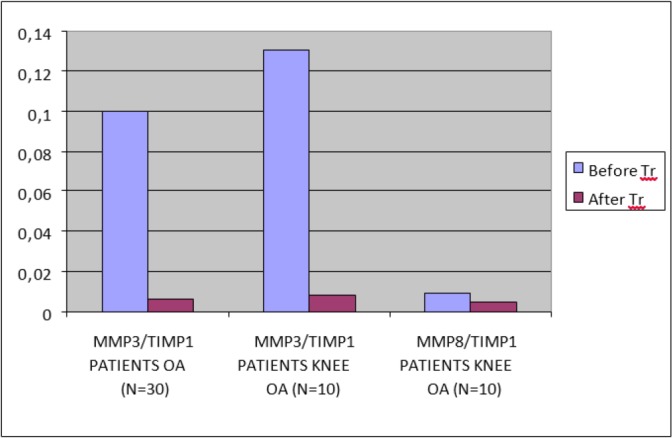
Effect of celecoxib on ratios MMP-3/TIMP-1 and MMP-8/TIMP-1

When the ratios MMP-1/TIMP-1, MMP-3/TIMP-1 and MMP-8/TIMP-1 were estimated in serum of the whole study group, a significant reduction was seen only in 220 MMP-3/TIMP-1 ratio after celecoxib administration (**Table 1, Figure 2**).

In order to estimate the effect of celecoxib on the levels of MMPs and TIMP-1 according to various locations of OA, we evaluated the same parameters in the three subgroups of patients. **Table 1** and **Figure 2** illustrate a significant reduction concerned the ratios MMP-3/TIMP-1 (p=0,036) and MMP-8/TIMP-1 (p=0,049) in the subgroup of 16 patients suffered from knee OA. In the subgroup of hip OA, a significant reduction of MMP-3 (p=0,011, **Table 1**, **Figure 1**) was noticed after the end of treatment with celecoxib. In hand OA, the effect of celecoxib MMPs and TIMP and the ratios of MMPs to TIMP was not significant.

### Effect of meloxicam on serum levels of MMPs and TIMP-1

**Figure 3.**
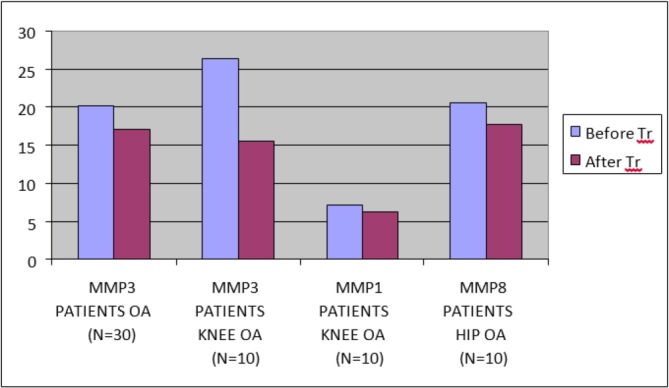
Effect of meloxicam on MMP-1 MMP-3 and MMP-8

The administration of meloxicam for all 36 patients included in our study resulted in a significant reduction only in MMP-3 levels (p=0,047) as showed in **Table 2** and **Figure 3**. Specifically for the knee OA cases, there was a reduction in the MMP-1 levels (p=0,044) and MMP-3 levels (p=0,005) as presented (**Table 2**, **Figure 3**). Furthermore, the MMP-3/TIMP-1 ratio was reduced to statistically significant levels at the end of treatment (p=0,017, **Table 2**). In addition, in the subgroup of hip OA patients, MMP-8 levels (p=0,005, **Table 2, Figure 3**) as well as MMP-8/TIMP-1 ratio (p=0,003, **Table 2**) showed significant differences after treatment. On the contrary, for the hand OA patients, the mean concentrations of MMPs and TIMP-1 were not significantly changed in all 36 patients of our study.

**Table 2.** Effect of meloxicam on MMPs and TIMP-1 levels in OA

	**Patients with OA**			
	**knee OA (n=16)**	**hip OA (n=10)**	**hand OA (n=10)**	**total (n=36)**

MMP-1^a^ BT	7,10	3,57	4,63	5,43
MMP-1 AT	6,2	3,74	4,80	5,14
MMP-1/TIMP-1^b^ BT	0,003	0,001	0,001	0,003
MMP-1/TIMP-1 AT	0,002	0,001	0,002	0,002

MMP-3^a^ BT	26,32^*^	19,83	10,54	20,13^*^
MMP-3 AT	15,46	23,06	13,59	17,05
MMP-3/TIMP-1^b^ BT	0,13	0,007	0,004	0,002
MMP-3/TIMP-1 AT	0,007	0,008	0,007	0,001

MMP-8^a^ BT	14,96	20,61^*^	17,29	14,40
MMP-8 AT	15,48	17,58	12,18	15,15
MMP-8/TIMP-1^b^ BT	0,007	0,007^*^	0,002	0,005
MMP-8/TIMP-1 AT	0,007	0,005	0,006	0,006

TIMP-1^a^ BT	313,12	264,00	247,00	281,11
TIMP-1 AT	228,12	261,00	225,00	235,69

* : p<0,01

a : Serum concentrations in ng/ml

b : Ratio

BT: Before treatment

AT: After treatment

### Effect of aceclofenac on serum levels of MMPs and TIMP-1

In patients treated with aceclofenac, the levels of MMPs and TIMP-1 were not significantly changed in OA subgroups.

In this study, it was also examined whether the order of NSAIDs administration was capable to affect the values of the parameters. It was revealed that a change in the order of administration of the three NSAIDs did not affect the final values of MMPs and TIMP-1 or their corresponding ratios after the completion of treatment.

**Table 3** shows the results obtained from the comparisons of the mean values of MMP-1, MMP-3, MMP-8 and TIMP-1 before and after treatment with all three selected anti-inflammatory drugs. From all comparisons, it was observed that only the mean concentration of MMP-3 was significantly reduced (p=0,02) after the end of treatment with all selected NSAIDs. This observation concerns the total of 36 patients and the subgroup of knee OA patients (p=0,001).

**Table 3.** Effect of celecoxib, meloxicam and aceclofenac.

	**Patients with OA**			
	**knee OA (n=16)**	**hip OA (n=10)**	**hand OA (n=10)**	**total (n=36)**

MMP-1^a^ BT	5,35	4,00	4,59	4,76
MMP-1 AT	5,56	4,00	5,13	5,00

MMP-3^a^ AT	26,26^*^	18,12	12,01	20,04^*^
MMP-3 AT	13,81	12,00	11,12	12,54

MMP-8^a^ BT	16,50	24,44	9,30	16,71
MMP-8 AT	12,60	20,00	10,02	14,00

TIMP-1^a^ BT	223,75	260,00	237,00	237,51
TIMP-1 AT	228,75	266,00	249,00	244,72

* : p<0,01

a : Serum concentrations in ng/ml

BT: Before treatment

AT: After treatment

In general, the mean MMP-3 concentrations were reduced after celecoxib and meloxicam treatment while aceclofenac did not reduced MMP-3 values.

## DISCUSSION

Currently, there is no curative treatment for OA, and no drug is proven to affect the morphological changes in articular cartilage. Therefore, it is essential to evaluate drugs which used until now to treat OA according to their effect on articular cartilage. Non-steroidal anti-inflammatory drugs (NSAIDs) are the most widely used drug therapy in OA. However, it remains controversial as to what effects these agents have on the progression of the disease. Preliminary studies in vitro or in vivo showed that some NSAIDs had favorable or neutral effect on the progression, but others had detrimental effect on it, and very few clinical trials have been carried out.^21^

However, in OA there are no reliable biochemical markers for diagnosis, evaluating disease severity or therapeutic response. Consequently, the research on biochemical markers in serum or synovial fluid could be very useful in OA.

In clinical trials concerning OA, biochemical assays of molecular markers have been promoted as prognostic markers, as serum levels of hyaluronan and Comp (cartilage oligometric protein). Especially, molecular markers were used reflected the increased bone turnover as specific deoxypiridoline cross links in urine, serum osteocalcin and bone sialoprotein.^29,30^ Recently newly developed specific and sensitive biochemical markers reflecting the cartilage turnover have been developed: type II collagen C-telopeptide fragments in urine (CTX-II) and urinary excretion of glucosyl galoctosyl pyridinoline (Glc-Gal-PYD).^31,32^

A few clinical trials showed that levels of specific MMPs in serum and synovial fluid could be used as markers of joint damage progression.^33,34^ However, existing clinical trials, which concerned a few number of patients, are restricted and the MMPs’ future practical use remains to be proven. In OA, the slowly progressive degeneration of cartilage could be estimated by the degree of joint-space narrowing (JSN) in X-Rays. However routine radiology to establish JSN is fraught with problems in reproducible measurements and measurable change may require clinical trials of 2–3 years duration with very large numbers of patients. In the past decade, magnetic resonance imaging (MRI) has emerged as a possible diagnostic technique to assess cartilage degeneration and to detect early signs of OA, but its wide use is confined especially for practical and economical reasons.^29,35^

This study aims at contributing in the quest for sensitive and appropriate markers for OA structural lesions. The study set out to determine the levels of metalloproteinases MMP-1, MMP-3 and MMP-8, as well as tissue inhibitor TIMP-1 in patients with osteoarthritis, following the administration of anti-rheumatic agents. Furthermore, the goal of the study was to identify and assess the possible effect of anti-rheumatic agents on the level of MMPs and TIMP-1 – given that most studies to date have been in vitro, very few in vivo and even fewer performed at a clinical level – taking into account the metabolism of articular cartilage. Additionally, the study aimed at investigating the possible contribution of said agents in clinical practice, so as to evaluate therapeutic outcome.

During the celecoxib administration MMPs concentration levels and MMPs/TIMP-1 ratios were investigated for all 36 patients included in the study. Comparison of levels before treatment and after its completion with the specific NSAID revealed that only MMP-3 showed values that were low enough to be statistically significant after the treatment. Furthermore, a significant reduction was noted in the MMP-3/TIMP-1 ratio, which resulted not only from the MMP-3 reduction, but also from a slight increase in TIMP-1. These findings indicate an effect of celecoxib on MMP-3, a metalloproteinase that is considered to be of importance for the degeneration of articular cartilage.

Celecoxib is a special COX-2 inhibitor with proven anti-inflammatory action, both in OA and other types of inflammatory arthritis; it is also known for its protective action on the gastro-intestinal mucosa.^36,37^

However, according to international literature, its action on articular cartilage has not yet been clearly defined. Most studies have been performed in vitro and a few in vivo, while there are no clinical studies examining the relation of celecoxib administration with the level of MMPs. Indeed, Mastbergen et al. showed in vitro that under inflammatory conditions, celecoxib has a positive indirect effect on the level of proteoglycans of cartilage matrix, thus reducing COX-2 – enzyme activity.^38^ It was also found in in vitro cultures of human articular cartilage, that the effect of celecoxib increased the hyaluronane and proteoglycans.^39^ However, a recent study on the effect of NSAIDs on special chondrocyte cultures showed that celecoxib does not seem to affect MMP-3 production, while it was also found that IL-1β production was inhibited.^40^

Tindall et al. investigated the effect of a 12month course of treatment with celecoxib on the progress of OA, evaluating the joint space stenosis on patients with knee and hip OA. They concluded that long-term treatment with this NSAID did not particularly affect the progress of the disease nor did it accelerate the destruction of articular cartilage in OA.^41^

The selective action of celecoxib on MMP-3 levels in our own study could be explained by the important role of this enzyme in cartilage metabolism. MMP-3 is capable not only of breaking down many of the elements of the intracellular matrix, such as proteoglycans, gelatins, laminin, fibrinonectin, and type III, IV, IX and X collagen, but also of triggering the activation of other MMPs, such as MMP-1, MMP-7, MMP-8, MMP-9 and MMP-13.^42^ Increased serum levels of MMP-3 have been found in PA as well as OA, but at lower concentration levels. This finding has been correlated with the degree of joint destruction and with disease activity in RA.^43,44^ Naito et al. studied the levels of MMPs and TIMP-1 in patients with OA; they indicated that plasma MMP-3 levels were higher as compared to those of healthy controls, and that the increase was higher in patients with generalized OA as compared to patients with knee OA.^33^

Similar observations were seen in the hip OA; in the knee OA group of patients only MMP-3/TIMP-1 and MMP-8/TIMP-1 ratios were reduced by statistically significant levels, although MMP-3 levels were not affected.

Furthermore, existing literature concerning the investigation of MMPs considers the ratio of each MMP to TIMP-1, as a more sensitive determinant which can better express the disturbed balance between MMPs and TIMP-1 and indicate changes occurring in articular cartilage.^33,44^ When meloxicam, which is a selective COX-2 inhibitor, was administrated, significant reduction in MMP-3 levels was found in OA patients at the end of treatment. Furthermore, concentration levels of MMPs were different in the three types of OA location. So, in the group with knee OA, MMP-1 and MMP-3 levels as well as MMP-3/TIMP-1 ratio were reduced to statistically significant levels, while in the hip OA group, MMP-8 group and MMP-8/TIMP-1 ratio were reduced to statistically significant levels. Tissue inhibitor TIMP-1 levels did not change significantly. These findings could, to a certain extent, agree with relevant literature available; however, in the latter, the effect of meloxicam on articular cartilage and MMPs was investigated in vitro and no other clinical trial has been carried out yet. Thus, according to the paper by Sadowski and Steinmeyer, meloxicam reduced in vitro the levels of MMP-1 transcription, while the levels of MMP-3 and TIMP-1 were not affected.^45^ Furthermore, Blot et al. found that meloxicam increases the synthesis of proteoglycans and hyaluronane in vitro.^46^

Of all NSAIDs, aceclofenac, one of the selective COX-2 inhibitors seems to have been investigated most in international literature in regard to its effect on articular cartilage in both in vitro and in vivo studies. In our own study, aceclofenac did not significantly affect concentration levels and ratios of MMPs and TIMP-1, whether these were calculated for the number of patients or the three particular patient groups with knee, hip and hand arthritis, respectively. Specific findings do not fully agree with in vitro studies, which showed a positive effect of aceclofenac on articular cartilage. So, according to the study by Dingle et al., aceclofenac belongs to the type of NSAIDs that stimulate the synthesis of proteoglycans in the inter-cellular matrix; this action might well be due to inhibition of the activity of IL-1 in vitro, in the presence of a growth factor (IGF-1).^47^

The capability of aceclofenac to inhibit the action of IL-1 as well as TNF-α was supported by Gonzalez et al., in a study during which aceclofenac was administered to OA patients with high levels of cytokines in their blood.^48^ However, the knowledge on the direct effect of aceclofenac on metalloproteinases is limited and has been derived from in vitro studies with contradictory results. The study by Sanchez et al., in particular, which concerns the effect of certain NSAIDs on the metabolism of human chondrocyte culture, showed that aceclofenac enhanced the increase of concentration levels of glucosaminoglycans, by inhibiting IL-1β and IL-6, without affecting the production of MMP-3.^40^ On the contrary, another study performed on experimental models, showed that the administration of 4-hydroxyaceclofenac, i.e. the main metabolite of aceclofenac in humans, led to a reduction in the concentration levels of precursor forms of MMP-1 and MMP-3 in chondrocyte cultures.^49^

An important finding from the present study concerns the statistically significant reduction in MMP-3 levels at the end of treatment with all NSAIDs administrated; this reduction was not affected by the sequence of agent administration. In the particular patient groups, MMP-3 showed statistically significant reduction only in knee OA cases. This finding could well be attributed to the fact that the knee joint comprises a large surface of strong cartilage, which allows more biochemical measurements. Furthermore, this is a joint that bears the body weight and, therefore, is subject to significant mechanical loads. This mechanical factor, in combination with other parameters, such as heredity, obesity, intense physical activity and others, may contribute towards more intense degeneration of the inter-cellular matrix of the cartilage, resulting in the production of MMPs.

Celecoxib and meloxicam seem to affect MMP levels more in the total number of patients and in certain OA cases, such as knee and hip OA. Of the MMPs studied, MMP-3 is the most sensitive to these changes. In fact, MMP-3 levels were significantly reduced after treatment with NSAIDs. This finding indicates the importance of MMP-3 in monitoring the therapeutic treatment of our OA patients. On the contrary, tissue inhibitor TIMP-1 values were not affected to a statistically significant extent with any of NSAIDs administered. The differences in MMP levels in the three groups might be due to the different participation of parameters studied in these groups.

## CONCLUSIONS

Conclusions derived from this study may be summarized as follows:

1. Of the metalloproteinases studied, MMP-3 concentrations were significantly reduced after treatment with NSAIDs. Therefore, determining the level of MMP-3 in the serum of OA patients could be a useful marker of the therapeutic effect at cartilage level.
2. Celecoxib and meloxicam have an advantage when it comes to reducing MMP-3, MMP-1 and MMP-8, while aceclofenac seems to have a relatively neutral effect on these parameters. The concentration of TIMP-1 was not particularly affected by any of the agents administered.
